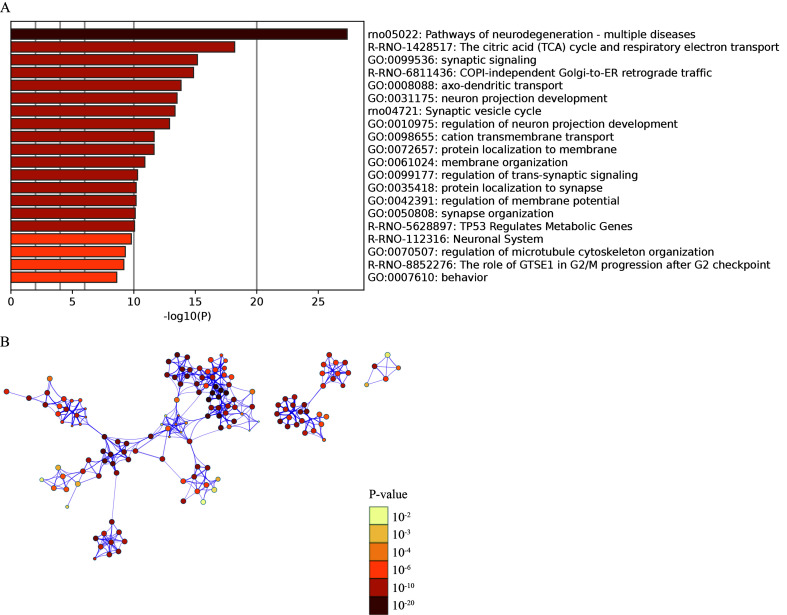# In situ tissue profile of rat trigeminal nerve in trigeminal neuralgia using spatial transcriptome sequencing

**DOI:** 10.1097/JS9.0000000000001110

**Published:** 2024-01-25

**Authors:** Wenbin Wei, Yuemin Liu, Yifen Shen, Tao Yang, Yabing Dong, Zixiang Han, Yiwen Wang, Zhiyang Liu, Ying Chai, Mengjie Zhang, Hanshao Wang, Hao Shen, Yihang Shen, Minjie Chen

**Affiliations:** a Department of Oral Surgery, Shanghai Ninth People’s Hospital, Shanghai Jiao Tong University School of Medicine; College of Stomatology, Shanghai Jiao Tong University; National Center for Stomatology; National Clinical Research Center for Oral Diseases; Shanghai Key Laboratory of Stomatology, Shanghai; b Central Laboratory; c Clinical Laboratory, Suzhou Ninth People’s Hospital; d Department of Medical Cosmetology, Suzhou, Jiangsu, People’s Republic of China

**Keywords:** myelin sheath, spatial transcriptomics sequencing, trigeminal ganglion, trigeminal neuralgia

## Abstract

**Background:**

Trigeminal neuralgia (TN) is the most common neuropathic disorder in the maxillofacial region. The etiology and pathogenesis of TN have not been clearly determined to date, although there are many hypotheses.

**Objective:**

The goal of this study was to investigate the interactions between different types of cells in TN, particularly the impact and intrinsic mechanism of demyelination on the trigeminal ganglion, and to identify new important target genes and regulatory pathways in TN.

**Methods:**

TN rat models were prepared by trigeminal root compression, and trigeminal nerve tissues were isolated for spatial transcriptome sequencing. The gene expression matrix was reduced dimensionally by PCA and presented by UMAP. Gene function annotation was analyzed by Metascape. The progression of certain clusters and the developmental pseudotime were analyzed using the Monocle package. Modules of the gene coexpression network between different groups were analyzed based on weighted gene coexpression network analysis and assigned AddModuleScore values. The intercellular communication of genes in these networks via ligand–receptor interactions was analyzed using CellPhoneDB analysis.

**Results:**

The results suggested that the trigeminal ganglion could affect Schwann cell demyelination and remyelination responses through many ligand–receptor interactions, while the effect of Schwann cells on the trigeminal ganglion was much weaker. Additionally, ferroptosis may be involved in the demyelination of Schwann cells.

**Conclusions:**

This study provides spatial transcriptomics sequencing data on TN, reveals new markers, and redefines the relationship between the ganglion and myelin sheath, providing a theoretical basis and supporting data for future mechanistic research and drug development.

## Graphical abstract

**Figure:**
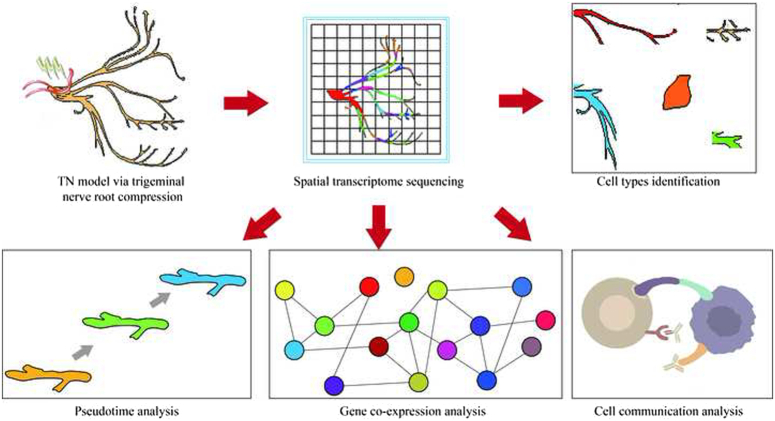

## Introduction

Highlights

- Definition of cell type-specific clusters in trigeminal nerve of rat trigeminal neuralgia (TN) model.
- Dynamic changes of gene expression in ganglion before and after TN occurrence.
- Demyelination and remyelination of Schwann cells in TN.
- Intercellular communication between myelin and trigeminal ganglion.

Trigeminal neuralgia (TN) is a common nerve disease characterized by episodic facial pain, affecting 182 out of every 100 000 people, and it often occurs in middle-aged and elderly individuals^1,2^. The internal medicine treatment of TN requires the long-term use of analgesics to control or alleviate pain, most commonly carbamazepine and oxcarbazepine. The mechanism primarily involves blocking voltage-gated sodium ion channels to stabilize hyperexcited neural membranes and inhibit competitive discharge^3^. Additionally, it can be used in combination with other medications, such as lamotrigine, baclofen, pregabalin, and gabapentin, but they are not effective in treating persistent pain and may have significant side effects, such as liver damage, bone marrow suppression, rash, drowsiness, gastrointestinal reactions, and ataxia^4^. For some patients, surgical treatment is needed because long-term medication use results in poor pain relief or toxic side effects. Currently, there are two types of surgery, classified by surgical site^5,6^: intracranial and extracranial. However, surgery is suitable only for specific types of TN, is not universally effective, and involves a high degree of difficulty, unstable therapeutic effects, and potential complications that may cause more serious nerve damage and sensory loss than the TN itself.

The 1-year overall recurrence rate for medication and surgical treatments can be as high as 10–50%. As the population ages, the incidence of TN is increasing annually. Moreover, effective etiological therapy is lacking in clinical practice^7,8^. Therefore, in-depth exploration and clarification of the pathogenesis of TN, as well as the search for important regulatory pathways and key target sites, have great clinical value and far-reaching application prospects.

Despite decades of research, no theory can fully explain the clinical symptoms of TN. The most accepted theory is peripheral nerve damage with demyelination, which causes short circuiting between adjacent axons and consequent pain^9^. This damage can be caused by vascular compression, bony compression, or abnormal discharge. Similar to peripheral nerve lesions, central nervous system lesions, including damage to dopaminergic neurons in the black substance of the striatum, increased excitability of the trigeminal sensory nucleus and reticular structure in the brainstem, and lesions in the trigeminal spinal nucleus, can also cause TN. Immune and inflammatory responses mediated by macrophages, mast cells, and vascular endothelial cells within the nerves can also cause neuropathic pain of the trigeminal nerve^10^. Neuropeptides associated with pain, such as substance P^11^ and calcitonin gene-related peptide (CGRP)^12^, can also cause pain by weakening the activity of monoamine neurotransmitters and endogenous opioid systems. Finally, abnormalities in sodium and calcium ion channel function can increase cell membrane excitability, raise action potential firing frequency, and generate pain signals^13^.

In TN, numerous cell–cell communications among trigeminal nerve cells, trigeminal nerve and immune cells^14,15^, stromal fibroblasts^16^, myelin Schwann cells^17^, endothelial progenitors^18^, etc., and the consequent molecular signaling participate in orchestrating the sensitization of nociceptive pathways, with alterations in ion channels, activation of immune cells, glial-derived mediators, and epigenetic regulation^19^. However, this complicated intercellular connection has never been systematically studied.

To investigate the interactions between different types of cells in the TN, particularly the impact and intrinsic mechanism of demyelination on the trigeminal ganglion, and to discover new important target genes and regulatory pathways in the TN, spatial transcriptomics sequencing was performed on a rat model to determine the expression profiles of different states and tissues in the TN, focusing on the RNA and functional enrichment involved in changes in the trigeminal ganglion, myelin sheath tissue, and nerve roots. The analysis also identified changes in transcripts throughout the disease development process, elucidated relationships involved in multigene coexpression, and derived cell–cell interactions. For the first time, this study provides spatial transcriptomic sequencing data on TN, reveals a set of new markers, and redefines the relationship between myelin sheaths and ganglion, providing a theoretical basis and data support for future mechanistic research and drug development.

## Methods

### Animal study

Five adult male Sprague–Dawley rats (Shanghai Jiao Tong Laboratory Animal Center) weighing 200–220 g were randomly allocated into two cohorts: one group was subjected to chronic compression of the trigeminal nerve root (*n*=10), while the other performed sham operation without compression was served as healthy controls (HCs) (*n*=10). The TN model was established using a previously described approach^20,21^. Five days after the establishment of the TN model, measurements of the mechanical withdrawal threshold testing and face grooming were obtained using previously described methods^18,20,22–24^.

For mechanical withdrawal threshold testing, animals were placed in a testing enclosure and allowed to move their heads freely. They were acclimated for 30 min before a series of von Frey filaments (Cat. No. 514009, Shanghai Yuyan Instruments Co., Ltd.) were applied to their vibrissa pad. A positive response was considered when the animal withdrew its head upon stimulation, which was tested five times. A positive result required at least three positive responses. The minimum force needed to elicit a response was defined as the withdrawal threshold. Target force was measured by grams.

Facial grooming episodes, including ipsilateral, contralateral, or bilateral grooming, were recorded via a video camera, and the video recording was initiated after the rats adapted to the test cage for 20 min. An independent observer, blinded to the treatment status of the rats, analyzed the face grooming behavior. The total time of asymmetrical face grooming episodes was noted for unilateral strokes with the dominant paw during each of the 10 min observation sessions. The work was reported in accordance with the Animals in Research: Reporting of In Vivo Experiments (ARRIVE) guidelines^25^ (Supplemental Digital Content 1, http://links.lww.com/JS9/B759).

After behavioral tests stabilized, the rats were sacrificed, and trigeminal nerve tissues were sampled for consequent experiments.

### Spatial transcriptomic sequencing

Freshly isolated trigeminal nerve tissues (HC, *n*=2; TN, *n*=3) without fixation were embedded in OCT, rapidly cooled in liquid nitrogen, and then cryosectioned to a thickness of 10 μm using a CryoStar NX70 cryostat (Thermo Fisher Scientific, Waltham) at -20°C. The tissue sections were layered onto a Visium Spatial Tissue Optimization Slide containing oligonucleotides for mRNA capture (10× Genomics) as previously described^26^. Each capture area includes 5000 spatially barcoded oligonucleotides with a diameter of 55 μm and a center-to-center distance of 100 μm, covering 6.5 mm by 6.5 mm. A Master Mix, consisting of reverse transcription reagents and fluorescently labeled nucleotides, was added to the tissue sections to facilitate fluorescently labeled cDNA synthesis. Following enzymatic removal of the tissue, fluorescent cDNA covalently linked to the oligonucleotides on the slide was left behind. The visualization of fluorescent cDNA was verified under fluorescence imaging conditions using the Visium Imaging Test Slide, during which the optimal permeabilization time for maximum fluorescence signal and minimal signal diffusion was determined by comparing H&E and fluorescence images. The libraries were sequenced with paired-end 150 bp sequencing using the NovaSeq 6000 platform.

The Space Ranger platform was utilized to identify the tissue capture areas on the slide and differentiate reads for each spot based on spatial barcode information. The sample quality was assessed using STAR, which assessed the total number of spots, number of read pairs in each spot, number of detected genes, and unique molecular identifier counts. Data normalization was performed using Sctransform, which constructed a regularized negative binomial model of gene expression and identified high variance characteristics. Uniform Manifold Approximation and Projection (UMAP) was utilized for dimension reduction, while K-means was used to cluster the same type of spots together. Seurat was employed to identify genes with spatial expression tendencies via unsupervised clustering methods or based on prior knowledge. Cell types were identified utilizing the SPOTlight deconvolution algorithm for non-negative matrix factorization. Differentially expressed genes (DEGs) were identified using the MAST difference test, with adjusted *P*-values <0.001. Gene Ontology (GO) analysis was performed using ‘Metascape’ to elucidate the functions and associated signaling pathways of the DEGs.

### Immunofluorescence (IF) assay

As previously described^21^, frozen trigeminal nerve sections (20 μm) were cut by a cryostat (Thermo Fisher Scientific), placed into mounting solution (1× PBS 18.4 ml, BSA 0.2 g, 10% Triton X-100 800 μl, goat serum 800 μl) for 30 min, and then blocked with 5% horse serum in PBS for 1 h at 4°C. Primary antibody diluent (0.3% Triton X-100 75 μl, 1× PBS 24.75 ml, goat serum 250 μl) and secondary antibody diluent (0.3% Triton X-100 250 μl, 1x PBS 25 ml) were freshly prepared. Sections were incubated with primary antibodies against SEMA5A (1:300, Cat. No. PA5-30884, Thermo Fisher Scientific) and PLXNB3 (1:500, Cat. No. PA5-48065, Thermo Fisher Scientific) at 4°C overnight. The next day, the sections were rinsed three times with 1× PBS for 10 min each time. All subsequent operations were carried out in the dark. The sections were placed in a new 200 μl tube and incubated with secondary antibodies (Alexa Fluor 488-conjugated goat anti-rabbit, 1:5000, Cat. No. Ab150081, Abcam; Alexa Fluor 647-conjugated donkey anti-Sheep, 1:5000, Cat. No. Ab150179, Abcam) at room temperature for 1 h. After washing with 1× PBS six times, the sections were placed on an adhesive glass slide and placed in a light-proof humidifier to dry to avoid peeling off during the subsequent process. The sections were covered with DAPI (20 μl) for 20 min for nuclear staining. Then, the DAPI was removed, and the water around the tissue was absorbed. Next, antifluorescence quenching mounting tablets (10–20 μl/sheet) were added to each tissue, and the slides were mounted with a cover glass. The images were captured by a DM2000 fluorescence microscope (Leica Biosystems).

## Results

### Definition of cell type-specific clusters in the trigeminal nerve of the rat TN model

**Figure 1:**
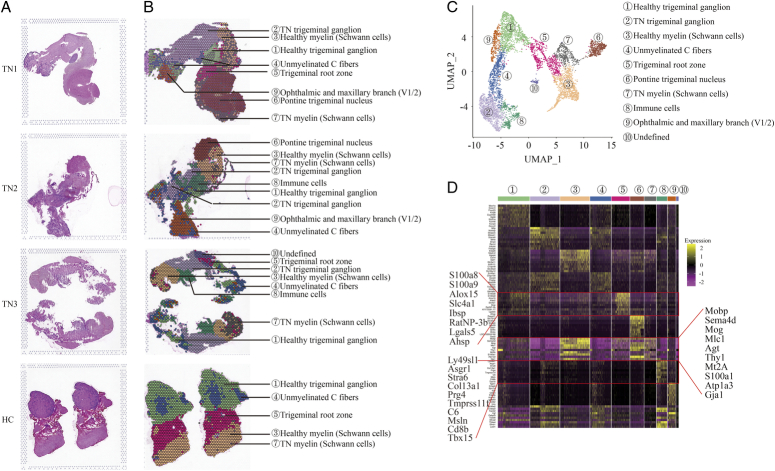
Characterization of cell clusters in rat trigeminal nerve tissues by spatial transcriptomics sequencing. (A, B) H&E staining (A) and feature plot (B) *in situ* showing the gene expression profiles of different parts of trigeminal nerve tissue of TN and HC. (C) UMAP classifying 10 clusters based on the transcriptomes of overall gene expression relationships among the 6773 spots with an average of 17 187 genes. (D) Heatmap showing the top 10 highly expressed genes in each cluster identified. Different cell clusters are color-coded. HC, healthy control; TN, trigeminal neuralgia.

The TN rat model was established and validated successfully by mechanical withdrawal threshold testing and face-grooming frequency behavior (Figure S1, Supplemental Digital Content 2, http://links.lww.com/JS9/B760). However, due to sample limitations, accurately capturing the entire trigeminal nerve in the designated area (6.5 mm×6.5 mm) of the Visium Spatial Gene Expression Slide posed a challenge. Thus, the trigeminal root, semilunar ganglion, and proximal branches from the diseased side of three individuals (TN, TN1, 2, 3) were isolated and squeezed into the first three capture areas. Meanwhile, the healthy side from two individuals was placed in the final capture area for HCs. After H&E staining (Fig. 1A), ten distinct global transcriptome clusters were identified (Fig. 1B, C), indicating good specimen quality and sequencing. The count of spots, pair reads in each spot, genes detected, mitochondrial gene ratio, and unique molecular identifiers all further confirmed the quality of the specimen and sequencing (Figure S2A-D, Supplemental Digital Content 3, http://links.lww.com/JS9/B761).

After analysis of both the pathological morphology and the highly expressed genes in each cluster (Fig. 1D, Figure S3, Supplemental Digital Content 4, http://links.lww.com/JS9/B762, Table S1, Supplemental Digital Content 5, http://links.lww.com/JS9/B763), a specific cell population was preliminarily assigned to each cluster. Cluster 6 (brown), which is dispersed in TN1 and TN2, corresponds to the pontine nucleus of the trigeminal nerve. Cluster 3 (bisque) and Cluster 7 (gray) merged together and extended outward along the nucleus pons. Moreover, both Cluster 3 and Cluster 7 showed robust expression of *Mobp*, *Sema4d*, *Mog*, *Mlc1*, *Agt*, *Thy1*, *Mt2A*, *S100a1*, *Atp1a3*, and *Gja1*, which are associated with the myelin sheath (Table S1, Supplemental Digital Content 5, http://links.lww.com/JS9/B763). Notably, gray spots of Cluster 7 were exceedingly rare in HC but abundant in TN1, TN2, and TN3. Therefore, Cluster 3 and Cluster 7 are defined as myelin sheath cells with normal and TN status, respectively.

Next, spots of Cluster 5 (magenta) enriched in TN1 and HC were thought to represent the trigeminal root zone. The rare occurrence of Cluster 5 in TN2 and TN3 was probably because it was surrounded and covered by myelin. In Cluster 5, transcripts of *S100a8*, *S100a9*, *Alox15*, *Slc4a1*, *Ibsp*, *RatNP-3b*, *Lgals5* and *Ahsp* were particularly abundant compared to all other clusters (Fig. 1D, Figure S3, Supplemental Digital Content 4, http://links.lww.com/JS9/B762, Table S1, Supplemental Digital Content 5, http://links.lww.com/JS9/B763). The *S100A* gene family is a prognostic and therapeutic biomarker of immune cell infiltration and the inflammatory state^27,28^ and participates in calcium signal propagation^29^. *Alox15* acts as an inflammatory suppressor^30^ and regulates cell ferroptosis against ROS and membrane potential^31^. Hence, mRNAs associated with inflammation and action potential appeared to be enriched in the naked trigeminal root.

Anatomically, the trigeminal ganglion closely follows behind the root. Deep staining by hematoxylin was observed in Clusters 1 and 2 compared to other regions, representing the abundant nuclei gathering (Fig. 1A). However, Cluster 2 (orchid) mainly existed in TN1, TN2, and TN3 and was nearly all replaced by Cluster 1 (spring green) in HC, indicating that Clusters 1 and 2 corresponded to the healthy and TN semilateral ganglia, respectively. Cluster 9 (chocolate), which occurred primarily in TN1 and TN2, was highly overlapped with ophthalmic and maxillary branches (V1, V2), in which the sensory nerve fibers of the trigeminal nerve mainly pass through. Interestingly, the spots of Cluster 4 (blue) were present in all cases and were widely distributed in the regions outward from the ganglion. On the other hand, a superposition of gene patterns was observed in which all highly expressed genes in Cluster 4 were also present in Cluster 1 or 2. However, it was difficult to define an intermediate state between normal and TN because Cluster 4 also appeared in HC. Therefore, it was tentatively identified as the unmyelinated C fibers.

Finally, spots of Cluster 8 (green) gathered at the ganglion in TN2 and TN3 showed the vigorous expression of *Fn1*, *Lyz2*, *Col13a1*, *Prg4*, *Ifitm1/2*, *Igfbp7*, *Emp1*, *C6* and *Bgn* (Fig. 1D, Figure S3, Supplemental Digital Content 4, http://links.lww.com/JS9/B762, Table S1, Supplemental Digital Content 5, http://links.lww.com/JS9/B763), which were connected with defense against bacteria, lysozyme enzyme activity, cell adhesion and migration, and complement and coagulation cascades. This cell population had an expression profile similar to the characteristic expression profile of immune cells. The rare spots of Cluster 10 (purple) that appeared only in TN3 were undefined, probably from a bone fragment given the expression of *Camk1* (regulating proliferation of calvarial osteoblasts)^32^. Overall, the potential cell population or different parts of the trigeminal nerve were initially defined for all clusters with distinct transcriptomic patterns in this system.

### Dynamic changes in gene expression in the ganglion before and after TN occurrence

**Figure 2:**
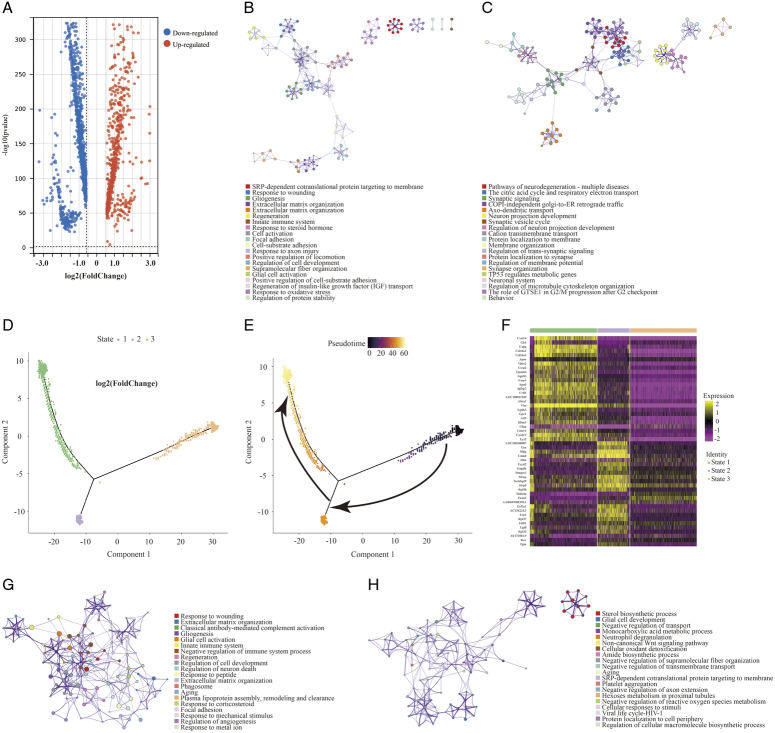
The differentially expressed genes (DEGs) and associated functions of the trigeminal ganglion in TN. (A) Volcano plot showing the DEGs (FC > 1.5 or < 0.67, *P* < 0.05) compared between trigeminal ganglion cell bodies (Cluster 2 vs. Cluster 1). (B, C) Enriched functional analysis of upregulated (B) and downregulated genes (C) from (A) by ‘Metascape’. (D, E) Scatter plots showing the evolution of cell subpopulations of the trigeminal ganglion by cell trajectory analysis (D) and pseudotime (E). (F) Heatmap showing the DEGs (FC > 1.5 or < 0.67, *P*<0.05) between State 1 and State 2 from (D). (G, H) Enriched functional analysis of upregulated (G) and downregulated genes (H) from (F) by ‘Metascape’.

Next, the DEGs of the ganglion cell body were analyzed (Cluster 2 vs. Cluster 1). In TN, a total of 674 genes in the ganglion were upregulated, while 1085 genes were downregulated (FC > 1.5 or < 0.67, *P* < 0.05) (Fig. 2A, Figure S4, Supplemental Digital Content 6, http://links.lww.com/JS9/B764, Table S2, Supplemental Digital Content 7, http://links.lww.com/JS9/B765). GO analysis showed that upregulated genes were enriched in the functions of SRP-dependent cotranslational protein targeting to membrane, response to wounding, gliogenesis, extracellular matrix organization, cell adhesion and so forth (Fig. 2B, Figure S5, Supplemental Digital Content 8, http://links.lww.com/JS9/B766), whereas downregulated genes were associated with neurodegeneration, citric acid cycle, respiratory electron transport, and synaptic organization (Fig. 2C, Figure S6, Supplemental Digital Content 9, http://links.lww.com/JS9/B767). The spatial trajectory assay indicated that spots representing the ganglion displayed two additional states in TN in the experimental system (Fig. 2D, Figure S7, Supplemental Digital Content 10, http://links.lww.com/JS9/B768). Pseudotemporal ordering suggested that Cluster 2 of the ganglion underwent a progressive pathological process (Fig. 2E). The DEGs of spots between these two branches were further analyzed (State 1 vs. State 2 in Fig. 2D), and a total of 159 upregulated and 76 downregulated genes (FC > 1.5 or < 0.67, *P* < 0.05) were observed in State 1 compared to State 2 of the ganglion (Fig. 2F, Table S3, Supplemental Digital Content 11, http://links.lww.com/JS9/B769). In State 1, a series of genes, such as *A2m*, *Jun*, *Fn1*, *Cd44*, and *C1qb*, induced inflammatory response, wound response, and complement activation via extracellular matrix organization (Fig. 2G). In turn, sterol/cholesterol biosynthetic and metabolic process were the top terms in pathway and process enrichment analysis in State 2 of the ganglion, with elevated expression of *Cyp51*, *Hmgcr*, *Fdft1*, *Insig1*, *Fdps*, *Idi1* and *Dhcr24* (Fig. 2H). In addition, compared to HC, the dynamic changes in the expression of numerous large and small ribosomal subunit proteins, *Tram1*, *Sec61b*, *Calr*, *Stx6*, *Hsp90b1* and *P4hb,* as ribosome, endoplasmic reticulum and Golgi apparatus biomarkers, were consistent across the entire cell trajectory diagram except for *Hmox2* (Figure S8, Supplemental Digital Content 12, http://links.lww.com/JS9/B770). SRP-dependent cotranslational transport^33^ appeared to play a vital role in modulating normal function or pain occurrence in the trigeminal nerve. In summary, the DEGs and related biological functions in the ganglion in TN were preliminarily described.

### Demyelination and remyelination of schwann cells in TN

**Figure 3:**
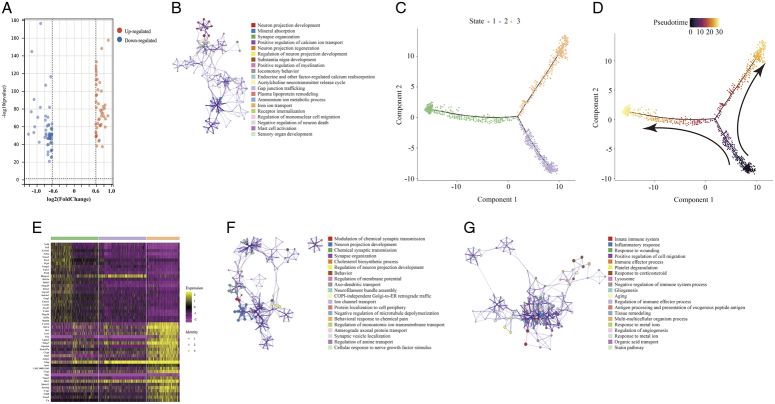
The differentially expressed genes (DEGs) and associated functions of myelin sheath Schwann cells in TN. (A) Volcano plot showing the DEGs (FC > 1.5 or < 0.67, *P*<0.05) compared between myelin sheath Schwann cells (Cluster 7 vs. Cluster 3). (B) Enriched functional analysis of DEGs from (A) by ‘Metascape’. (C, D) Cell trajectory analysis showing the evolution of cell subpopulations of the myelin sheath by cell state (C) and pseudotime (D). (E) Heatmap showing the DEGs (FC > 1.5 or < 0.67, *P*<0.05) between State 1 and State 3 from (C). (F, G) Enriched functional analysis of upregulated (F) and downregulated genes (G) from (F) by ‘Metascape’.

Between TN and HC, 106 DEGs in the myelin sheath were found (Fig. 3A, Table S4, Supplemental Digital Content 13, http://links.lww.com/JS9/B771). In addition to myelin sheath-related important regulatory proteins, such as *Myrf*, *Mag* and *Mog,* which were markedly compromised, the iron metabolism-related genes transferrin (*Tf*), ferritin (*Fth1*)^34^ and *Slc48a1* ^35^ were downregulated. On the other hand, *Atp2b2*, *Cabp1*, *S100a1*, *S100b*, *Rasgrp3*, *Pcp4*, *Vsnl1* and *Hpcal1,* which are involved in calcium homeostasis and signaling, were all significantly changed (Fig. 3A). Due to the limited number of differential genes, functional enrichment analysis was performed for upregulated and downregulated genes together. The exchange and transport of iron ions, calcium ions, and acetylcholine transmitters warrant further study of the role of myelin in TN (Fig. 3B). Similarly, myelin sheath Schwann cells in different TN cases exhibited two different statuses of pathological progress beyond HC (Fig. 3C, D, Figure S9, Supplemental Digital Content 14, http://links.lww.com/JS9/B772). A total of 138 upregulated and 137 downregulated genes (FC > 1.5 or < 0.67, *P* < 0.05) were found between State 1 and State 3 (Fig. 3E, Table S5, Supplemental Digital Content 15, http://links.lww.com/JS9/B773). Functional enrichment analysis showed that the upregulated genes corresponding to these two states implied cell differentiation, development, and regeneration (Fig. 3F) as well as cell senescence and apoptosis mediated by the inflammatory response (Fig. 3G). These two completely different functional gene sets indicated demyelination and remyelination in TN.

### Intercellular communication between myelin and the trigeminal ganglion

**Figure 4:**
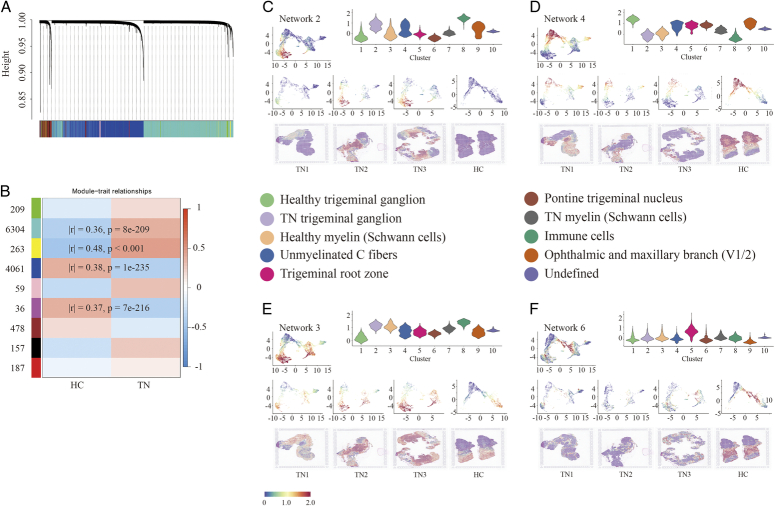
Coexpressed gene modules among all spots. (A) Gene clustering tree constructed by the dissTOM matrix using weighted correlation coefficients (top). The distribution of genes in nine modules with different colors (bottom). (B) Heatmap showing the module trait relationships calculated by Pearson correlation. The modules with |r| ≥ 0.3 and *P*<0.05 are highlighted. (C-F) Feature plots of Network 2 (C), Network (4), Network 3 (E), and Network 6 (F) *in situ* showing the scores of each gene module by AddModuleScore. Violin plot (top right) showing the scores of the gene module in each cluster.

To further investigate the cellular coordination and communication between ganglion and Schwann cells, weighted gene coexpression network analysis (WGCNA) was conducted to identify nine coexpression modules of a total of 11 754 genes within all spots by comparing TN 1/2/3 and HC (Fig. 4A, Table S6, Supplemental Digital Content 16, http://links.lww.com/JS9/B774). In particular, four networks displayed a significant correlation (|r| ≥ 0.3, *P*<0.05) (Fig. 4B), in which two networks containing 6304 and 263 genes were highly expressed, whereas the other two containing 4061 and 36 genes were reduced in TN compared to HC (Figure S10, Supplemental Digital Content 17, http://links.lww.com/JS9/B775). Notably, high expression of genes in Network 2 (turquoise) was particularly prominent in the trigeminal ganglion of TN compared to HC (Fig. 4C). In contrast, Network 4 (blue) contributed to the specific genotype in the healthy trigeminal ganglion (Fig. 4D). In addition to the ganglion and immune cells, the gene signature of Network 3 (yellow) was specific to myelin Schwann cells (Fig. 4E), whereas Network 6 showed the signature of the trigeminal root zone (Fig. 4F). Most of the coding genes were involved in Networks 2 and 4, suggesting that large transcriptomic changes occurred between healthy and diseased trigeminal ganglia. In contrast, few DEGs were found in myelin tissue or the trigeminal root zone.

**Figure 5:**
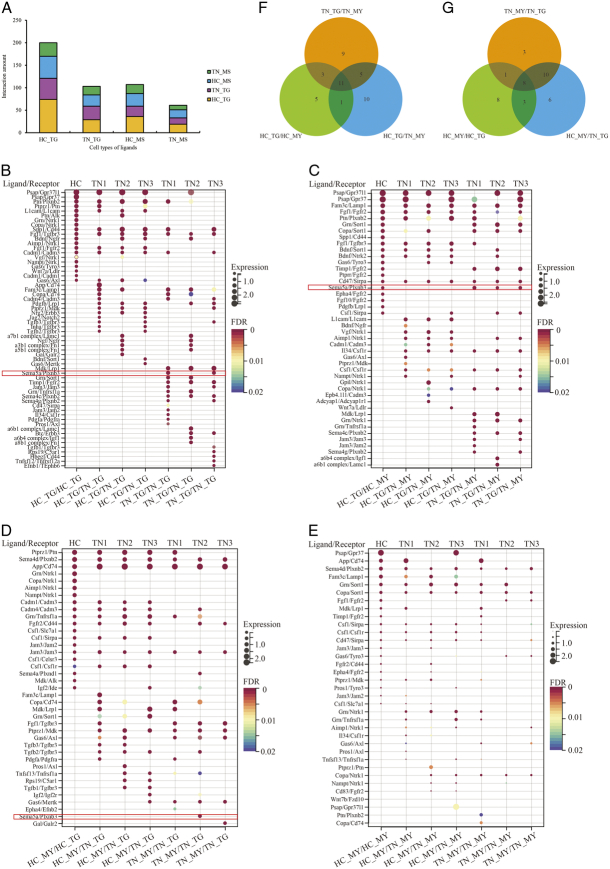
Intercellular communication between the trigeminal ganglion and myelin sheath. (A) Stacked column chart showing the number of ligand–receptor interactions across the trigeminal ganglion and myelin sheath in HC and TN. (B–E) Dot plots showing the specific intercellular communications in the trigeminal ganglion (B), from the trigeminal ganglion to the myelin sheath (C), from the myelin sheath to the trigeminal ganglion (D) and in the myelin sheath (E). The color of the circles indicates the level of significance, and the larger the circles are, the greater the gene expression value of the interacting cells. The top 20 pairs are selected. Sema5a/Plxnb3 is highlighted by a red frame. (F, G) Venn diagram highlighting the specific intercellular communications from the trigeminal ganglion to the myelin sheath (F) and from the myelin sheath to the trigeminal ganglion (G). HC, healthy control; MS, myelin sheath; TG, trigeminal ganglion; TN, trigeminal neuralgia.

**Figure 6:**
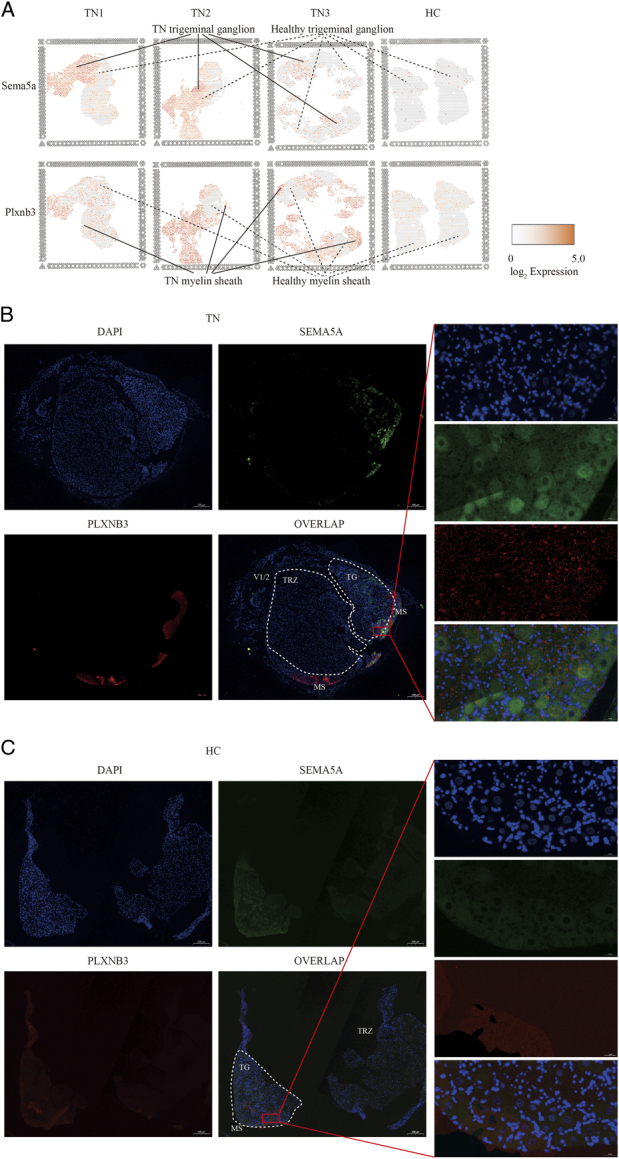
Expression of Sema5a/Plxnb3 in TN. (A) Feature plots showing the distribution of Sema5a (top) and Plxnb3 (bottom) positive expression spots in TN. (B, C) Immunofluorescence assay showing the expression of SEMA5A and PLXNB3 in TN (B) and HCs (C) at magnifications of 5× (left) and 80× (right). The trigeminal ganglion, myelin sheath, trigeminal root zone, and branches are highlighted by dashed lines in the overlapping image. HC, healthy control; MS, myelin sheath; TG, trigeminal ganglion; TRZ, trigeminal root zone; V1/2, ophthalmic and maxillary branches; TN, trigeminal neuralgia.

The intercellular communication of genes in these networks between trigeminal ganglion and myelin Schwann cells was further constructed in a ligand–receptor manner using CellPhoneDB analysis. It was observed that the healthy trigeminal ganglia and myelin sheath had more robust connections with each other than diseased ones (Fig. 5A). Unlike the self-interaction of the trigeminal ganglion in HC, the pairs *Psap*/*Gpr37*, *Grn*/*Ntrk1*, *Copa*/*Ntrk1*, *Aimp1*/*Ntrk1*, *Nampt*/*Ntrk1*, *Gas6*/*Tyro3*, *Wnt7a*/*Ldlr* and *Cadm1*/*Cadm1* were lost, whereas the pairs *Pdgfb*/*Lrp1*, *Ptprzl*/*Mdk*, and *Nrg2*/*Erbb3* did not appear in the normal ganglion but did appear in the diseased trigeminal ganglion in TN (Fig. 5B). Moreover, *Mdk*/*Lrp1*, *Timp1*/*Fgfr2* and *Sema4c*/*Plxnb2* appeared in the self-interaction of the diseased trigeminal ganglion (Fig. 5B). For intercellular communication between the trigeminal ganglion and myelin Schwann cells, the ligand–receptor pairs *Spp1*/*Cd44*, *Ptprr/Fgfr2*, *Epha4*/*Fgfr2*, *Fgf10*/*Fgfr2*, *Pdgfb*/*Lrp1*, *Grn/Ntrk1*, *Copa/Ntrk1*, *Aimp1/Ntrk1*, *Nampt/Ntrk1*, *Csf1/Slc7a1*, *Jam3/Jam2*, *Csf1/Celsr3* and *Mdk/Alk* only existed in HC (Fig. 5C, D). In turn, the responses to the trigeminal ganglion by healthy or diseased myelin sheaths were largely similar (Fig. 5D), indicating that the effects of the myelin sheath on the trigeminal ganglion were independent of the disease status. This conclusion could also be applied to the self-interactions of myelin Schwann cells (Fig. 5E). The presence of more specific ligand–receptor pairs from the trigeminal ganglion to the myelin sheath than from the myelin sheath to the trigeminal ganglion (Fig. 5F, G) suggested that the demyelination or remyelination of the myelin sheath was likely to be modulated by extracellular signals from the trigeminal ganglion. Finally, the ligand–receptor pair *Sema5a*/*Plxnb3* was considered as an example. This pair was primarily found in both the ganglion and myelin sheath of the TN (red frame highlighted in Fig. 5B–D). The spatial distribution of mRNA abundance (Fig. 6A) confirmed that the spots with positively expressed Sema5a and Plxnb3 were robust in TN compared to HC. IF assays also showed that the ligand–receptor pair SEMA5A/PLXNB3 was a TN-specific signature (Fig. 6B) compared to HC (Fig. 6C). However, the pattern of SEMA5A expression fit well with the nucleus of the trigeminal ganglion, indicating that it was seldom expressed in the myelin sheath (Fig. 6B). In contrast, PLXNB3 was extensively expressed in the trigeminal ganglion and myelin sheath. Here, it was speculated that the Sema5a/Plxnb3 connection was particularly involved in the self-interaction of the trigeminal ganglion (Fig. 5B) and in signaling from the trigeminal ganglion to the myelin sheath (Fig. 5C) but not from the myelin sheath to the trigeminal ganglion (Fig. 5D).

## Discussion

The greatest advantage of spatial transcriptome sequencing is the ability to match information of RNA molecules to a specific tissue area *in situ* ^36^. Differential mRNA expression profiles can accurately indicate the molecular divergence among different tissue/cell types as well as lesions and nonlesion tissues. Sometimes this advantage can be used to identify different tissue types that lack distinct histological boundaries, such as certain neuronal nuclei^26^.

However, current fabrication techniques do not allow spatial transcriptome resolution to reach the single-cell level, because each spot (55 μm diameter) can still accommodate 7–8 cells. In general, the spatial transcriptome in combination with single-cell sequencing can produce deep single-cell resolution transcriptome maps in tissues through deconvolution and mapping. Successful integration of single-cell and spatial transcriptome data analyses will contribute to understanding the structure of cell type distribution and the hypothesized mechanisms of cellular communication that underlie this structure.

Nevertheless, the trigeminal nerve is too large to allow the use of single-cell suspensions for RNA sequencing. In previous attempts, large amounts of cell debris were observed, suggesting that the axon and dendrite dissociated from the cell body, and RNA molecules were lost with the cytoplasm at the rupture. Without the support of single-cell sequencing, it is not possible to effectively evaluate the expression profile of each cell at single-cell resolution. However, this is not necessary for the trigeminal nerve. This study only used spatial transcriptome sequencing, which still provided sufficient information to uncover the cellular and molecular changes in TN.

The initial hypothesis is that the trigeminal neurons are so long that RNA or protein distribution may be uneven among the trigeminal nerve root, ganglia, and branches, providing rapid feedback regarding diseased areas. For example, *Robo2* is important in determining the sensory projection of V1/2 branches^37^, while *Pax3* is robustly expressed in the V1 branch^38^.

Numerous studies on phase separation have demonstrated that enzymes and their substrates that perform specific functions aggregate in specific spaces and times, forming an independent compartment with the ability to promote reaction occurrence and prevent interference from irrelevant substances in the cytoplasm, as exemplified in various membrane-bound organelles, such as the mitochondria, Golgi apparatus, and endoplasmic reticulum, in many eukaryotic cells^39–42^.

The transcriptomes of ganglion and ophthalmic and maxillary branches (Cluster 9 and Cluster 2) as well as ganglion and nerve roots (Cluster 1 and Cluster 5) were compared in the three TN samples, and DEGs were preliminarily obtained. The RNA synthesized from the trigeminal ganglion is believed to be differentially distributed in different areas, but the underlying mechanism of these differences is not clear.

In addition, the specimens collected in this study were partially covered by myelin sheath cells, and the transcriptome information in some areas is actually a superposition of the trigeminal nerve and the myelin sheath cells. The next step in this research is continued sample optimization. However, since the results on this topic are independent of other results, they are not presented in this article.

In the comparison of the normal and TN trigeminal ganglion, it was unexpectedly found that the upregulated genes in TN are more involved in regeneration, repair, extracellular matrix remodeling, and other related processes, while the downregulated genes are associated with various neurodegenerative diseases, oxidative respiratory chain, axonal connection transmission, etc., which suggests that the TN trigeminal ganglion exhibits abnormalities in aerobic metabolism and neural transmission function but nevertheless performs functions to improve this pathological state (Fig. 2B, C).

The spatial trajectory analysis further subdivided the trigeminal ganglion into two different pathological states, and the highly expressed genes in State 2 were involved in the inhibition of adverse reactions such as free radical and fibrosis. Notably, steroid synthesis dominates this biological process (Fig. 2H), and the use of steroid hormone therapy for TN has been confirmed by previous studies^43–45^. This indicates that State 2 corresponds to the state of self-repair of the trigeminal ganglion. In contrast, the biological processes involved in the highly expressed genes in State 1 indicate that the semilunar ganglion has undergone processes such as death, detachment, and structural remodeling, which are much more intense than those in State 2.

The changes in the myelin sheath are relatively simple: one side continuously demyelinates, while the other side generates new Schwann cells to reconstitute the node of Ranvier. Ferroptosis-related genes such as *Tf*, *Fth1*, and *Slc48a1* were found in demyelinating Schwann cells, which is a new discovery that may elucidate the molecular mechanisms of demyelination and has already been incorporated into future research plans.

The balance of calcium ions is also important for the function of the myelin sheath. Calcium ions play an important role in the formation and stability of the myelin phospholipid layer and can promote the mitosis of Schwann cells^46,47^.

After the identification of numerous DEGs, signal transmission between trigeminal neurons and Schwann cells surrounding the myelin sheath was investigated. Clarifying the causal relationship between these processes is important to explain the demyelination effect in TN.

First, the WGCNA analysis results showed a relatively small number of genes (only 263) with significant differences in the myelin sheath (Fig. 4E). Moreover, many other genes, such as *Atp2b2*, *Thy1*, *Atp1b1*, and *Cabp1*, related to the regulation of calcium homeostasis were found in Module 4, while *Plp1*, *Ramp3*, *S100a1*, and *Gjc2* were found in Module 7. This suggests that most of the gene functions in Schwann cells are related to the gene expression of trigeminal neurons, and only a small part truly participate in self-regulation.

Second, CellPhoneDB analysis confirmed that the ligand–receptor pairs from trigeminal neurons and Schwann cells are far more abundant than the pairs from Schwann cells to nerve cells (Fig. 5). The results showed preliminary evidence of one ligand–receptor pair, *Sema5a*/*Plxnb3,* in trigeminal neurons and myelin in TN (Fig. 6). This coexpression signature was verified by an IF assay. *Plxnb3*, as a receptor on neurons, receives *Sema5a* signals from both neurons and myelin, but the mechanism has not yet been elucidated. One previous study suggested that *Sema5a*/*Plxnb3* can induce the Warburg effect in liver cancer^48^, which implies that energy metabolism, especially glucose metabolism and oxygen utilization in mitochondria, may be one of the mechanisms causing TN.

In future studies, several important ligand–receptor pairs will be selected for in-depth study to test the hypothesis. In any case, the consensus that demyelination causes TN seems to need further discussion.

Overall, the limitations in this study included the following: the data of too small branches are not well collected, and the myelin sheath and nerve are bonded to each other, resulting in data overlap, which is the problem introduced by sampling. None of the findings in the analysis have been verified by cellular and molecular biology, although deeper investigations will be performed in future research.

## Conclusions

In summary, this study utilizes the latest technology to provide a batch of spatial transcriptome data sets on rat TN, providing extensive new information. The analysis identified the DEGs and related functions of the trigeminal ganglion and myelin Schwann cells in normal and TN and tentatively established a potential relationship between them (mainly in terms of ligand–receptor manner). The results may help to elicit more valuable responses or ideas from others in this field. Many important genes remain to be discovered and studied by researchers worldwide.

## Ethical approval

This study was approved by the Animal Care and Use Committee for Shanghai Ninth People’s Hospital (SH9H-2022-A168-SB).

## Consent for publication

Informed consent was obtained from all individual participants included in the study.

## Sources of funding

This project is funded by National Natural Science Foundation of China (82370981) Shanghai Science and Technology Commission (21Y11903500), the Ninth People’s Hospital Affiliated to Shanghai Jiao Tong University School of Medicine (JYHJB202204), Rare disease registration project of the Ninth People's Hospital Affiliated to Shanghai Jiao Tong University School of Medicine (JYHJB202204), Jiangsu Province High-level Innovation and Entrepreneurship Talent Introduction Plan (JSSCBS20222022), Suzhou Medical Health Science and Technology Innovation Project (SKYD2022023), Suzhou Science and technology plan (SKY2023032), Gusu Health Talent Training Project (GSWS2019087, GSWS2022111 Program of Developing Public Health through Science and Education of Wujiang District, Suzhou (wwk202306)). We are also grateful to Shanghai OE Biotech and GeneFund Co., Ltd. for sequencing data analysis, and Coweldgen Scientific Co. Ltd. for assistance of animal model construction.

## Author contribution

MSc W.W. and MSc L.Y. performed all animal experiments; MSc S.Y.F. conducted all biological experiments; PhD Y.T. prepared and organized all figures. They contributed equally to this work. MSc D.Y., MSc H.Z., MSc W.Y., MSc L.Z., MSc C.Y., MSc Z.M., and MSc W.H. assisted in animal experiments and biological analysis; MSc S.H., PhD S.Y.H, and PhD C.M. designed all preject, as well as drafted and revised manuscript. All authors read and approved the final manuscript.

## Conflicts of interest disclosure

The authors declare that they have no conflicts of interest.

## Research registration unique identifying number (UIN)

No human study.

## Guarantor

Yihang Shen.

## Presentation (for original articles only)

None.

## Availability of data and materials

Clean data was deposited in GEO database assigned with GSE233838.

## Provenance and peer review

Not commissioned, externally peer-reviewed.

## Supplementary Material

SUPPLEMENTARY MATERIAL

## Supplementary Material

**Figure:**
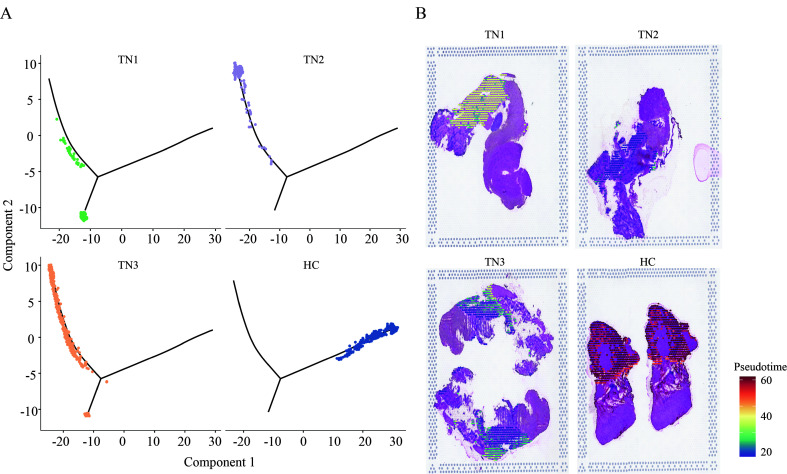


**Figure:**
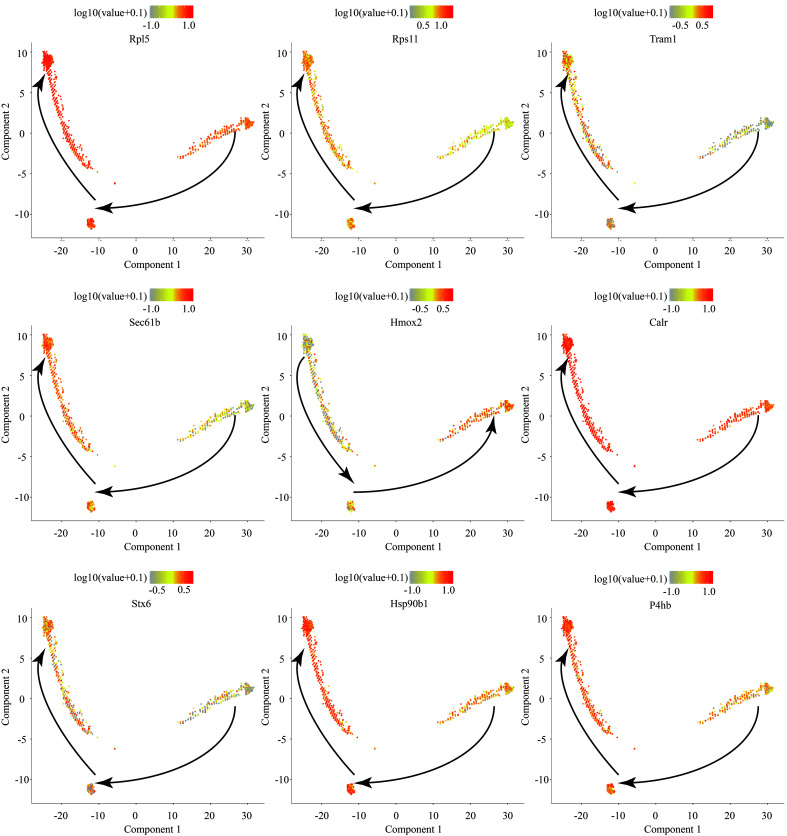


**Figure:**
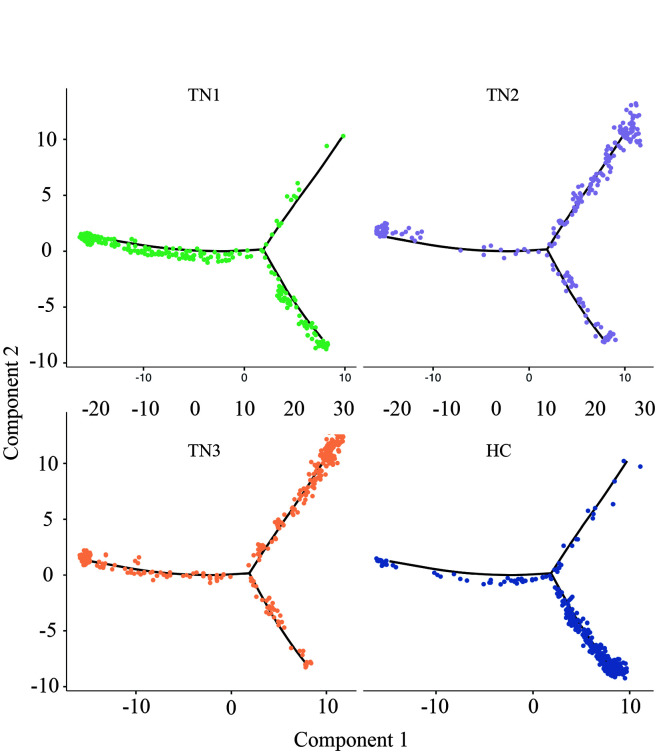


**Figure:**
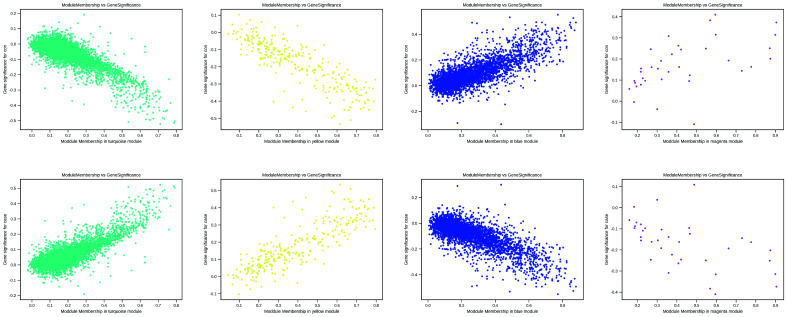


**Figure:**
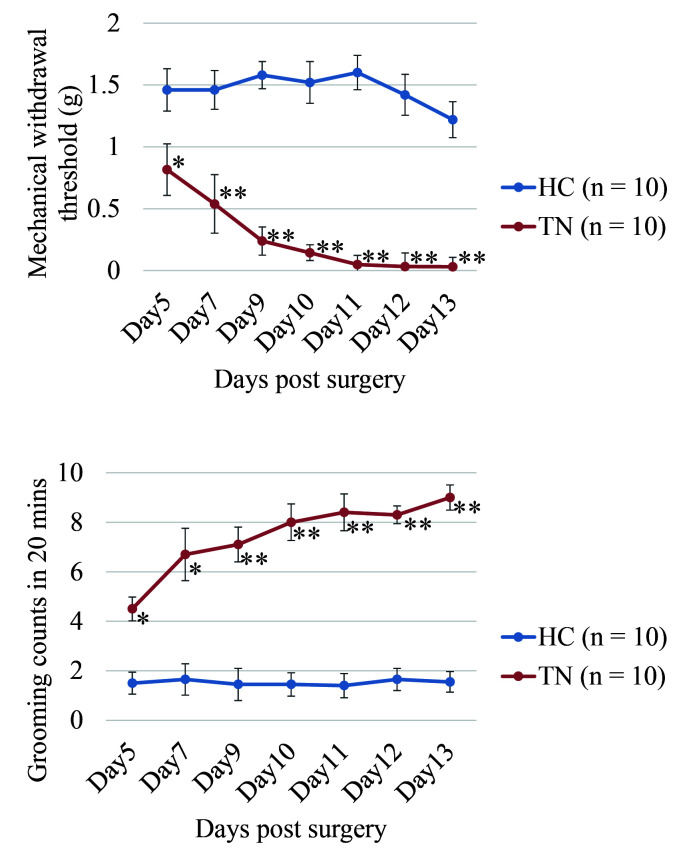


**Figure:**
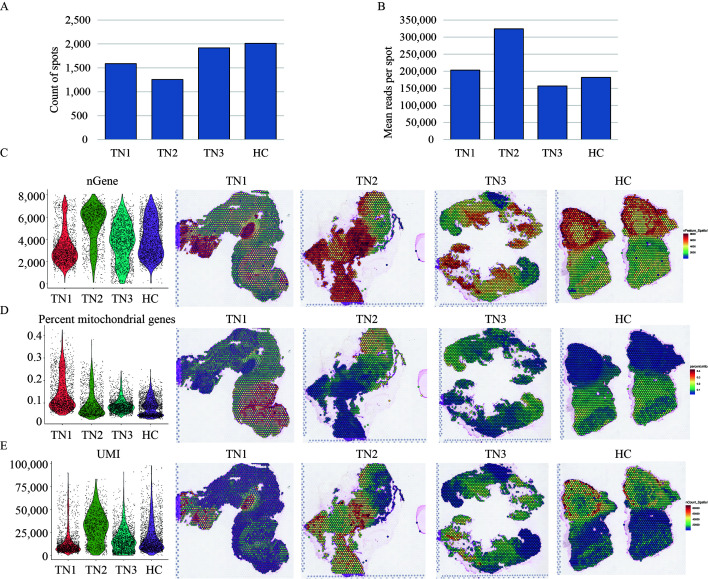


**Figure:**
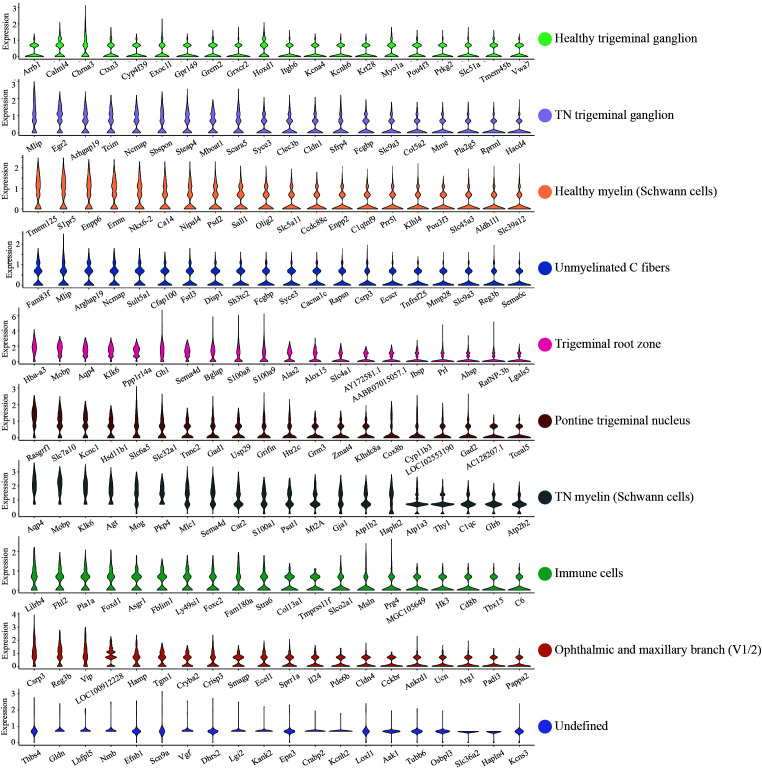


**Figure:**
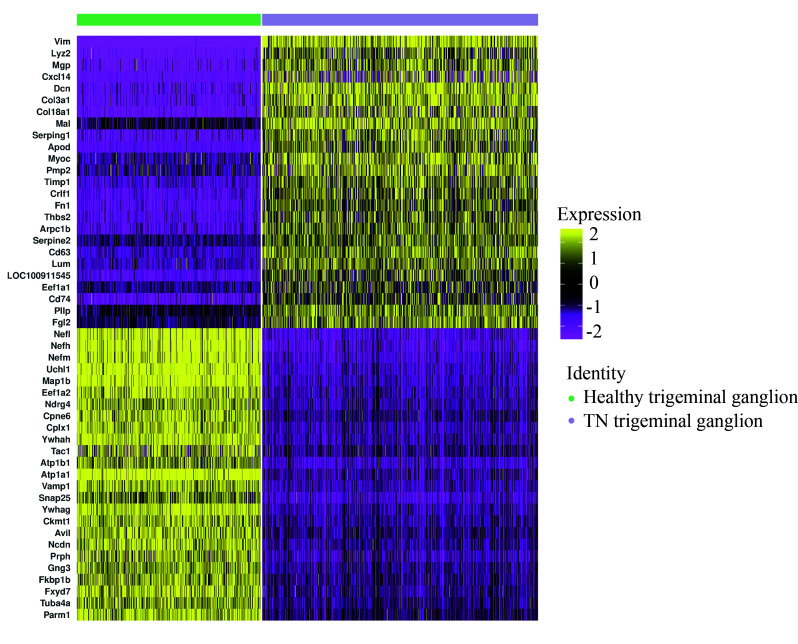


**Figure:**
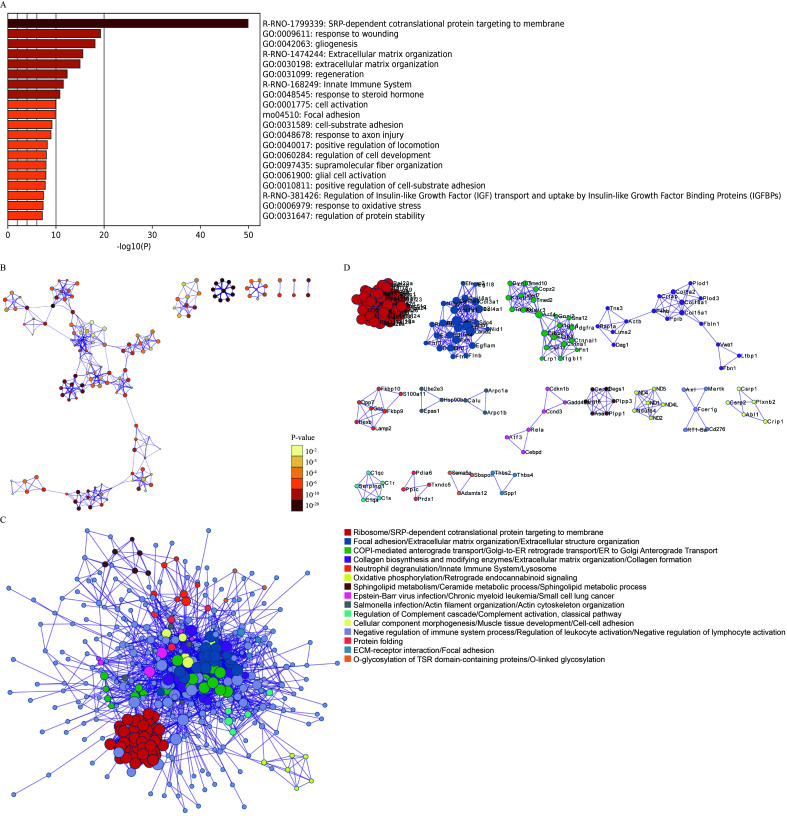


**Figure:**